# The first survey of the Saudi Acute Myocardial Infarction Registry Program: Main results and long-term outcomes (STARS-1 Program)

**DOI:** 10.1371/journal.pone.0216551

**Published:** 2019-05-21

**Authors:** Khalid F. Alhabib, Abdulhalim J. Kinsara, Saleh Alghamdi, Mushabab Al-Murayeh, Gamal Abdin Hussein, Shukri AlSaif, Hassan Khalaf, Hussam Alfaleh, Ahmad Hersi, Tarek Kashour, Ayman Al-saleh, Mohammad Ali, Anhar Ullah, Hassan Mhish, Abdulrahman Nouri Abdo, Fawaz Almutairi, Mohammed R. Arafah, Raed AlKutshan, Mubarak Aldosari, Basel Y. AlSabatien, Mohammad Alrazzaz, Adel M. Maria, Aziza H. Aref, Muhammed M. Selim, Ayman M. Morsy, Fathi A. AlTohari, Ammar A. Alrifai, Awatif A. Awaad, Hassan El-Sayed, Sherief Mansour, Ashraf A. Atwa, Salah Abdelkader, Naif Altamimi, Elnatheer Saleh, Wael Alhaidari, El Husseini A. ElShihawy, Ali H. Busaleh, Mohammed Abdalmoutaleb, Essam M. Fawzy, Zaki Mokhtar, Adil M. Saleh, Mohammed A. Ahmad, Adel Almasswary, Mohammed Alshehri, Khalid M. Abohatab, Turki AlGarni, Modaser Butt, Ibrahim Altaj, Farhan Abdullah, Yahya Alhosni, Hadia B. Osman, Najeebullah Bugti, Atif A. Aziz, Abdulrahman Alarabi, Ibrahim A. AlHarbi

**Affiliations:** 1 Department of Cardiac Sciences, King Fahad Cardiac Center, College of Medicine, King Saud University, Riyadh, Saudi Arabia; 2 Department of Cardiology, Ministry of National Guard Health Affair, King Saud bin Abdulaziz University for Health Sciences, COM-WR King Abdullah International Medical Research Center, Jeddah, Saudi Arabia; 3 Madinah Cardiac Center, Madinah, Saudi Arabia; 4 Armed Forces Hospitals Southern Region, Khamis Mushayt, Saudi Arabia; 5 King Salman (North West Armed Forces Hospital), Tabuk, Saudi Arabia; 6 Saud AlBabtain Cardiac Center, Dammam, Saudi Arabia; 7 Prince Sultan Cardiac Center, Buraydah City, Saudi Arabia; 8 Ha’il Cardiac Center, Hail, Saudi Arabia; 9 Saudi Heart Association, Riyadh, Saudi Arabia; 10 King Salman Heart Center, King Fahd Medical City, Riyadh, Saudi Arabia; 11 Obeid Specialized Hospital, Riyadh, Saudi Arabia; 12 Security Forces Hospital, Riyadh, Saudi Arabia; 13 Dallah Hospital, Riyadh, Saudi Arabia; 14 Aljazeera Hospital, Riyadh, Saudi Arabia; 15 King Saud Medical City (Riyadh Medical Complex), Riyadh, Saudi Arabia; 16 Alwatani /National Care Hospital, Riyadh, Saudi Arabia; 17 AlFalah Hospital, Riyadh, Saudi Arabia; 18 King Salman Hospital, Riyadh, Saudi Arabia; 19 Imam Abdulrahman Al Faisal Hospital, Riyadh, Saudi Arabia; 20 King Fahad General Hospital–Jeddah, Saudi Arabia; 21 AlNoor Specialist Hospital, Makkah, Saudi Arabia; 22 Security Forces Hospital, Makkah, Saudi Arabia; 23 King Abdulaziz Hospital and Oncology Center, Jeddah, Saudi Arabia; 24 Althager General Hospital, Jeddah, Saudi Arabia; 25 King Abdulaziz Specialist Hospital, AlTaif, Saudi Arabia; 26 AlZahra Hospital, Madinah, Saudi Arabia; 27 Al-Dar Hospital, Madinah, Saudi Arabia; 28 AlMiqat Hospital, Madinah, Saudi Arabia; 29 King Fahad Hospital, Madinah, Saudi Arabia; 30 Ohud Hospital, Madinah, Saudi Arabia; 31 Alansar Hospital, Madinah, Saudi Arabia; 32 Prince Sultan Cardiac Center, AlHofuf /AlHasa, Saudi Arabia; 33 Al Qateef Central Hospital, Al Qateef, Saudi Arabia; 34 Mohammad Dossary Hospital, Alkhobar, Saudi Arabia; 35 King Khalid General Hospital, Hafar Albatin, Saudi Arabia; 36 King Saud Hospital, Unizah, Saudi Arabia; 37 Dr.Sulaiman Alhabib Hospital, Buraydah, Saudi Arabia; 38 Alrass General Hospital, Arrass, Saudi Arabia; 39 Aseer Central Hospital, Abha, Saudi Arabia; 40 AlNamas General Hospital, AlNamas, Saudi Arabia; 41 Khamis Mushayt General Hospital, Khamis Mushayt, Saudi Arabia; 42 King Khalid Civilian Hospital, Tabuk, Saudi Arabia; 43 Arar Cardiac Center (Prince Abdulla bin Abdulaziz bin Musaed Cardiac Center), Arar, Saudi Arabia; 44 Prince Abdulaziz Bin Musaid Hospital, Arar, Saudi Arabia; 45 Prince Sultan Cardiac Center-King Khalid Hospital, Najran, Saudi Arabia; 46 Prince Meteb Ibn Abdulaziz Hospital, Sakaka, Saudi Arabia; 47 King Abdullaziz Specialist Hospital, Sakaka, Saudi Arabia; 48 Dumat AlJandal Hospital, Dumat AlJandal, Saudi Arabia; 49 King Fahd Hospital, AlBaha, Saudi Arabia; 50 King Fahad Central Hospital, Jizan, Saudi Arabia; Keele University, UNITED KINGDOM

## Abstract

**Background:**

Prior acute coronary syndrome (ACS) registries in Saudi Arabia might not have accurately described the true demographics and cardiac care of patients with ACS. We aimed to evaluate the clinical characteristics, management, and outcomes of a representative sample of patients with acute myocardial infarction (AMI) in Saudi Arabia.

**Methods:**

We conducted a 1-month snap-shot, prospective, multi-center registry study in 50 hospitals from various health care sectors in Saudi Arabia. We followed patients for 1 month and 1 year after hospital discharge. Patients with AMI included those with or without ST-segment elevation (STEMI or NSTEMI, respectively). This program survey will be repeated every 5 years.

**Results:**

Between May 2015 and January 2017, we enrolled 2233 patients with ACS (mean age was 56 [standard deviation = 13] years; 55.6% were Saudi citizens, 85.7% were men, and 65.9% had STEMI). Coronary artery disease risk factors were high; 52.7% had diabetes mellitus and 51.2% had hypertension. Emergency Medical Services (EMS) was utilized in only 5.2% of cases. Revascularization for patients with STEMI included thrombolytic therapy (29%), primary percutaneous coronary intervention (PCI); (42.5%), neither (29%), or a pharmaco-invasive approach (3%). Non-Saudis with STEMI were less likely to undergo primary PCI compared to Saudis (35.8% vs. 48.7%; respectively, p <0.001), and women were less likely than men to achieve a door-to-balloon time of <90 min (42% vs. 65%; respectively, p = 0.003). Around half of the patients with NSTEMI did not undergo a coronary angiogram. All-cause mortality rates were 4%, 5.8%, and 8.1%, in-hospital, at 1 month, and at 1 year, respectively. These rates were significantly higher in women than in men.

**Conclusions:**

There is an urgent need for primary prevention programs, improving the EMS infrastructure and utilization, and establishing organized ACS network programs. AMI care needs further improvement, particularly for women and non-Saudis.

## Introduction

The Global Burden of Diseases, Injuries, and Risk Factors Study, 2017, estimated that the largest number of deaths within non-communicable diseases (17·8 million; 95% uncertainty interval [UI]: 17·5–18·0 deaths) was due to cardiovascular diseases [1]. On a global level, between 2007 and 2017, deaths from ischemic heart disease increased from 7.30 million (95% UI: 7·22–7·46) to 8.93 million (95% UI: 8·79–9·14) [1]. Registry studies play a key role in improving the health care of patients that present with acute myocardial infarction (AMI) by assessing their real-life clinical presentation, management, and outcomes. The health care set-up in Saudi Arabia has several independent sectors that makes it very challenging to conduct a nation-wide registry, particularly due to lack of a unified system for triaging patients with suspected AMI. In previous local and regional registry studies, we have demonstrated that Saudi patients with acute coronary syndromes (ACSs) present at a relatively young age, due to the high prevalence of coronary artery disease (CAD) risk factors [2–4]. However, many medical centers enrolled in those registries were tertiary-care hospitals with robust health care infrastructures. In addition, there was an under-representation of some health care sectors, particularly the non-tertiary care Ministry of Health hospitals, private health care hospitals, and hospital in the peripheral areas of the country that are usually under-served and more in-need of improved care than hospitals in major cities. Moreover, those registries could have missed enrollment of a large proportion of non-Saudi expatriates, a subgroup with different demographic and socioeconomic backgrounds and with limited access to certain tertiary health care centers. Non-Saudis represent 37% of the 33 million people in the total population of Saudi Arabia [5].

The Saudi Acute Myocardial Infarction Registry (STARS) planned to overcome the limitations of previous registry studies by following a simple, yet unique and representative, snap-shot study design. The French Registry of Acute ST-Elevation or non-ST-elevation Myocardial Infarction (FAST-MI) study enrolled consecutive patients with AMIs every 5 years to create a representative sample of the healthcare centers in France, since 2005 [6]. That particular study methodology included multiple iterations, which resulted in a representative, reliable snap-shot of AMI care in that large European country. We sought to follow a similar study design in Saudi Arabia to include a sample that fully represented AMI care and long-term outcomes in a country with a vast geographic area that included several health care sectors. We report here the first survey of the registry program (STARS-1 Program).

## Methods

### Study design and population

This prospective, multi-center, STARS study included all consecutive hospital admissions of patients with AMI. Eligible patients were ≥18 years-old and were hospitalized with or without a ST-elevation myocardial infarction (STEMI or NSTEMI, respectively). Clinical assessments were performed at out-patient clinic visits or, when not possible, in phone calls at one-month and one-year follow-ups.

Eligible hospitals had a 50-bed capacity or more. In addition, over a one-month period, a minimum of 20 patients with AMI had to be enrolled from hospitals with no catheterization laboratories (non-Cath Lab hospitals), and a minimum of 60 patients with AMI had to be enrolled from hospitals with catheterization laboratories (Cath Lab hospitals). Hospitals were allowed to continue patient enrollment over another two months, when the assigned target number of patients was not achieved over the one-month period. Certain months were randomly assigned by a Principal Coordinating Center (PCC) for patient enrollment in the non-Cath Lab hospitals, and these were different from the months assigned to the Cath Lab hospitals. These separate enrollment periods were implemented to avoid the double-entry of patients transferred between the two types of hospitals, and to reduce the potential biases associated with patient enrollment in specific months in some hospitals (i.e., summer vacations or religious holidays). To improve the representation of the health care in this study, we planned to enroll two-thirds non-Cath Lab hospitals and one-third Cath Lab hospitals. In addition, the recruitment of hospitals from each region into the registry was based on the “real-life” relative distribution of the various health care sectors in Saudi Arabia.

This STARS Program survey aimed to provide a snap-shot of patient enrollment and geographic health care representation. The program will be repeated every five years to provide a reliable assessment of temporal changes in AMI care over the years in Saudi Arabia.

### Study objectives and definitions

This study aimed to assess the clinical presentations, management, in-hospital course, in-hospital mortality rates, and one-month and one-year re-admission and mortality rates of patients with AMI. In addition, we assessed the guideline-recommended medical treatments and the proportion of patients with uncontrolled CAD risk factors at the one-year follow-up. We employed standard definitions of these clinical variables, based on the American College of Cardiology and the European Society of Cardiology guidelines [7,8]. Accordingly, the availability of serum troponin data (rather than the creatinine kinase-MB fraction) was a mandatory requirement for hospital enrollment into the registry to ensure a highly reliable diagnosis of patients with NSTEMI.

### Study organization

A Registry Steering Committee, consisting of experienced cardiologists from all five major geographic regions of the country, was responsible for recruiting hospitals, monitoring the study progress in their respective regions, overlooking the overall study conduct, and approving the final study results. Throughout the hospital stay of each patient, a Case Report Form (CRF) was completed online by dedicated research assistants, physicians, and/or trained nurses working in each hospital. A log book was maintained to ensure enrollment of all consecutively admitted patients. All CRFs were verified by a cardiologist, then sent electronically to the PCC (King Fahad Cardiac Center, King Saud University, and Riyadh). There, the forms were checked for data queries or entry mistakes by a dedicated research coordinator and the Principal Investigator, before submission for final analysis. Ethics approval was obtained from the Institutional Review Board (IRB), College of Medicine, King Saud University, Riyadh, Saudi Arabia. Given the observational nature of the study and the fact that patient identities remained anonymous, the IRB did not require written informed consent.

### Statistical methods

Categorical data were summarized with absolute numbers and percentages. Continuous data were summarized as the means and standard deviations (SDs) or the medians and interquartile ranges (IQRs). Comparisons of categorical variables between groups were evaluated with the chi-square test or Fisher’s exact test. Comparisons of continuous data between groups were evaluated with the student t-test or Mann Whitney U test. Crude and adjusted odds ratios with 95% confidence intervals (CI) were estimated using logistic regression models, where adjustments were done for clinically relevant factors S5 and S6 Tables. All analyses were performed with SAS version 9.2 (SAS Institute, Inc, Cary, NC) and R software (R foundation for statistical computing, Vienna, Austria).

## Results

### Enrolled Hospitals and Study population

Between May 2015 and January 2017, 2233 patients with AMI were enrolled in the study. Of these, 1471 (65.9%) patients had STEMI and 762 (34.1%) patients had NSTEMI. We identified 370 eligible hospitals, and we invited a total of 50 hospitals (14%) from the 13 provinces in the country. 30 centers were non-Cath Lab hospitals (60%). The various health care sectors represented in the study were similar to those included in the overall health care system in Saudi Arabia (S1 and S2 Figs). Table 1 shows patient baseline characteristics, clinical presentation, and cardiac procedures. The mean age (±SD) of the overall population was 56 (±13) years, 55.6% were Saudi citizens, and 85.7% were men. Of the non-Saudis, 68.7% were of Arab ethnicity. The prevalence of CAD risk factors was high; 52.7% of patients had diabetes mellitus, 51.2% had hypertension, and 51.3% were either current or ex-smokers. The mean body mass index was 28.43 (±5.4) kg/m^2^. At presentation, only 5.2% were transferred by the Emergency Medical Services (EMS; i.e., Saudi Red Crescent); 88.3% had typical chest pain, and 16% had clinical evidence of congestive heart failure.

**Table 1:** Baseline characteristics, clinical presentation, and cardiac procedures in patients with acute Myocardial infarction.

Characteristic	Total N = 2233	STEMI N = 1471(65.88%)	NSTEMI N = 762(34.12%)	P-value
Age, Mean ± SD	55.79 ± 13.03	54.46 ± 12.56	58.37 ± 13.53	<0.001
Male, n (%)	1913 (85.67%)	1315 (89.39%)	598 (78.48%)	<0.001
Saudis, n (%)	1218 (55.6%)	759 (51.60%)	459 (60.24%)	<0.001
Ethnicities of non-Saudis, n (%)				
Arab	1533 (68.65%)	951 (64.65%)	582 (76.38%)	<0.001
South Asian	633 (28.35%)	473 (32.15%)	160 (21.00%)	
Others	67 (3.00%)	47 (3.20%)	20 (2.62%)	
BMI, Mean ± SD	28.43 ± 5.40	28.13 ± 5.27	29.01 ± 5.59	0.063
Type of STEMI, n (%)				
Anterior	779 (52.96%)	779 (52.96%)		
Inferior	553 (37.59%)	553 (37.59%)		
Other	139 (9.45%)	139 (9.45%)		
Medical History, n (%)				
Angina	471 (21.09%)	201 (13.66%)	270 (35.43%)	<0.001
Myocardial infarction	295 (13.21%)	120 (8.16%)	175 (22.97%)	<0.001
PCI	238 (10.66%)	99 (6.73%)	139 (18.24%)	<0.001
CABG	55 (2.46%)	15 (1.02%)	40 (5.25%)	<0.001
Heart failure	111 (4.97%)	32 (2.18%)	79 (10.37%)	<0.001
Stroke	81 (3.63%)	40 (2.72%)	41 (5.38%)	0.001
Chronic renal failure	121 (5.42%)	48 (3.26%)	73 (9.58%)	<0.001
Diabetes Mellitus	1177 (52.71%)	711 (48.33%)	466 (61.15%)	<0.001
Hypertension	1144 (51.23%)	649 (44.12%)	495 (64.96%)	<0.001
Hypercholesterolemia	761 (34.08%)	437 (29.71%)	324 (42.52%)	<0.001
Current/ex-smoking	1145 (51.28%)	794 (53.98%)	351 (46.06%)	<0.001
Chief complaint, n (%)				
Chest pain	1972 (88.31%)	1348 (91.64%)	624 (81.89%)	<0.001
Shortness of breath/Fatigue	132 (5.91%)	41 (2.79%)	91 (11.94%)	
Epigastric/shoulder/back/neck pain	96 (4.30%)	65 (4.42%)	31 (4.07%)	
Cardiac arrest	12 (0.54%)	9 (0.61%)	3 (0.39%)	
Others	21 (0.94%)	8 (0.54%)	13 (1.71%)	
First Medical Contact before presenting to hospital, n (%)	788 (35.29%)	598 (40.65%)	190 (24.93%)	<0.001
Emergency Department	688 (87.31%)	521 (87.12%)	167 (87.89%)	0.781
Clinic/Doctor	137 (17.39%)	106 (17.73%)	31 (16.32%)	0.655
Pharmacy	11 (1.40%)	7 (1.17%)	4 (2.11%)	0.339
Transferred by Saudi Red Crescent	96 (5.2%)	74 (5.03%)	22 (2.89%)	0.018
Status upon hospital arrival				
Heart rate, Mean ± SD	85.50 ± 21.21	85.42 ± 22.04	85.65 ± 19.51	0.810
Systolic blood pressure, Mean ± SD	133.1 ± 28.12	130.7 ± 28.41	137.6 ± 26.99	<0.001
Heart rate>100 bpm, n (%)	383 (17.15%)	248 (16.86%)	135 (17.72%)	0.610
Systolic blood pressure<90mmHg, n (%)	68 (3.05%)	57 (3.87%)	11 (1.44%)	0.002
Cardiac arrest	58 (2.60%)	50 (3.40%)	8 (1.05%)	<0.001
Heart Failure Killip Class, n (%)				
Class I	1876 (84.01%)	1260 (85.66%)	616 (80.84%)	<0.001
Class II/III	305 (13.66%)	168 (11.42%)	137 (17.98%)	
IV	52 (2.33%)	43 (2.92%)	9 (1.18%)	
Echo-Options, n (%)				
Normal LV systolic function (EF >50%)	616 (30.85%)	326 (25.08%)	290 (41.61%)	<0.001
Mild LV systolic dysfunction (EF 40–50%)	683 (34.20%)	467 (35.92%)	216 (30.99%)	
Moderate LV systolic dysfunction (EF 30–40%)	488 (24.44%)	364 (28.00%)	124 (17.79%)	
Severe LV systolic dysfunction (EF <30%)	210 (10.52%)	143 (11.00%)	67 (9.61%)	
Elective coronary angiogram (performed or Transferred with referral), n (%)	986 (44.16%)	578 (39.29%)	408 (53.54%)	<0.001
Arterial access, n (%)				0.765
Femoral	155 (48.44%)	3 (42.86%)	152 (48.56%)	
Radial	165 (51.56%)	4 (57.14%)	161 (51.44%)	

Abbreviations: STEMI: ST-elevation myocardial infarction, NSTEMI: non-ST-elevation myocardial infarction, BMI: body mass index, PCI: percutaneous coronary intervention, CABG: coronary artery bypass graft, LV: left ventricular, EF: ejection fraction.

Compared to patients with STEMI, those with NSTEMI were more likely to be older, Saudi citizens, and have a history of angina, myocardial infarction, PCI, CABG (Coronary Artery Bypass Grafting), heart failure, chronic renal failure, diabetes, hypertension, and/or hypercholesterolemia (Table 1). However, compared to patients with NSTEMI, patients with STEMI were more often men, current or ex-smokers, and presented with typical ischemic chest pain or cardiac arrest.

### ST elevation myocardial infarction: presentation and management

Among the 1178 patients with STEMI that presented within 24 h of symptom onset, 88.2% presented within 12 h of symptoms onset. The median time from symptom onset to the first medical contact was 110 min (IQR: 199.5), and the median time from the first medical contact to emergency department arrival was 155 min (IQR: 280; Fig 1).

**Fig 1:**
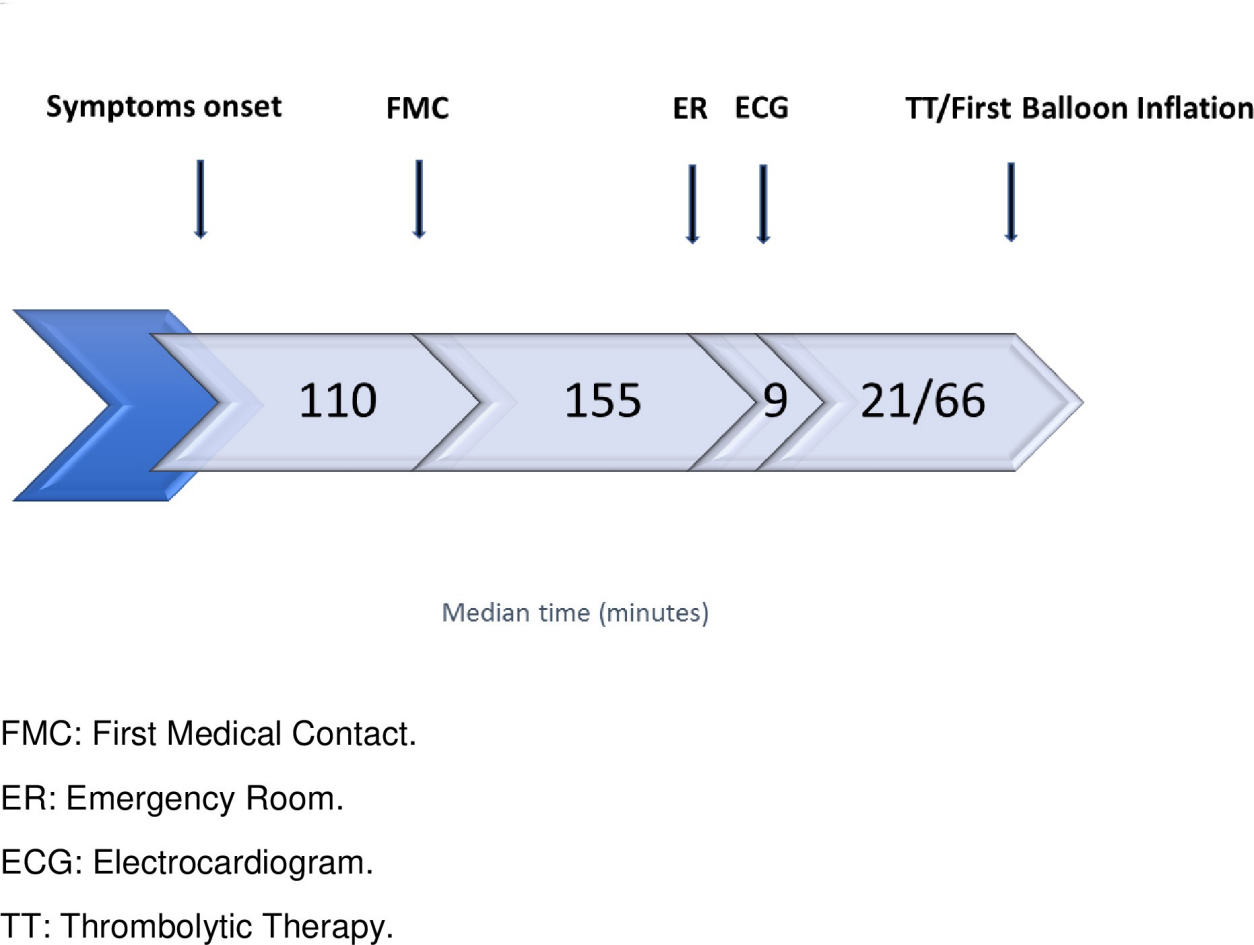
Time-line of patients with ST-segment elevation myocardial infarction. FMC: First medical contact; ER: emergency room; ECG: electrocardiogram; TT: thrombolytic therapy.

On the other hand, among patients who were self-presented to the emergency department, the median time from symptom onset to emergency department arrival was 180 min (IQR: 310). A total of 427 patients (29%) were treated with thrombolytic therapy; 22% had unsuccessful clinical reperfusions; of these, only 38% underwent rescue PCI. Administration of thrombolytic therapy followed by elective coronary angiography within 24 h in patients that experienced a successful reperfusion or rescue PCI in case of unsuccessful reperfusion (i.e., pharmaco-invasive approach) was performed in only 3% of patients. Primary PCI was performed in 42.5% of patients with STEMI. Among all PCIs, 48.2% were performed through a radial artery approach (50.4% in men vs. 29.3% in women, p = 0.002), and 19% were treated with aspiration thrombectomy devises. Around one-third of all patients with STEMI did not receive either thrombolytic therapy or a primary PCI, mainly due to a late presentation (53%).

The median door-to-needle time was 30 min (IQR: 35 min) with no significant difference between men and women. The median door-to-balloon time was 74 min (IQR: 84 min), and it was significantly longer in women (103 min, IQR: 196 min) than in men (73 min, IQR: 74 min; p = 0.033). However, door-to-needle times <30 min were achieved in 45% of patients, with no significant difference between men and women. Door-to-balloon times <90 min were achieved in 62% of patients, but women were less likely than men to achieve that benchmark (65% in men vs. 42% in women, p = 0.003; S1 and S2 Tables).

Around half of the patients with STEMI were non-Saudi. Thrombolytic therapy was used more frequently in non-Saudis than in Saudis (40.6% vs. 18.2%; respectively, p = <0.001). On the other hand, Saudis were more likely to receive primary PCIs than non-Saudis (48.7% vs. 35.8%; respectively, p <0.001).

### In-hospital medications

**Fig 2:**
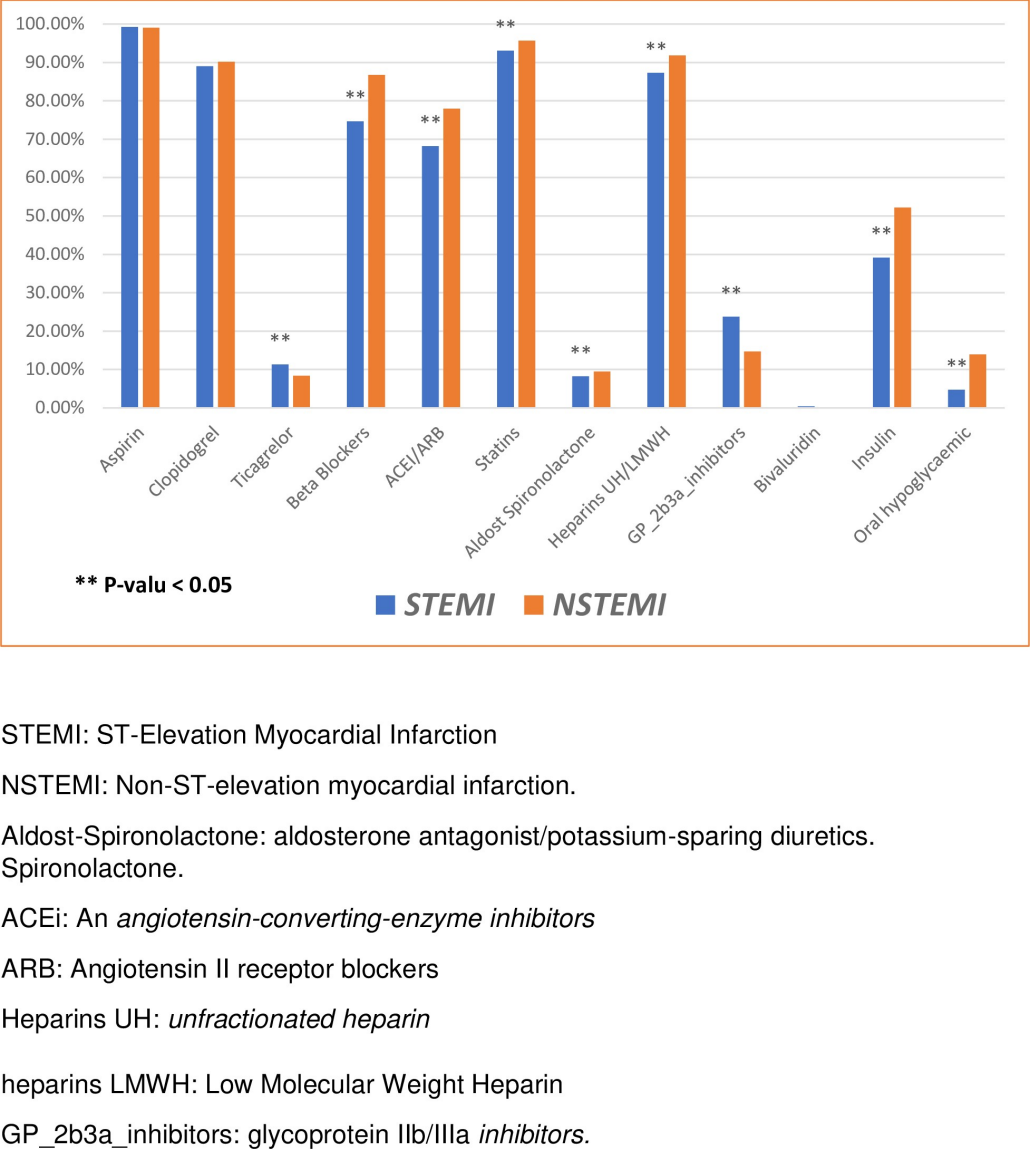
Medications use in the first 24 hrs of hospital admission for patients with acute myocardial infarction. STEMI: ST-elevation myocardial infarction; NSTEMI: non-ST-elevation myocardial infarction.

A high frequency of guideline-recommended treatments was given upon hospital admission Fig 2.

**Fig 3:**
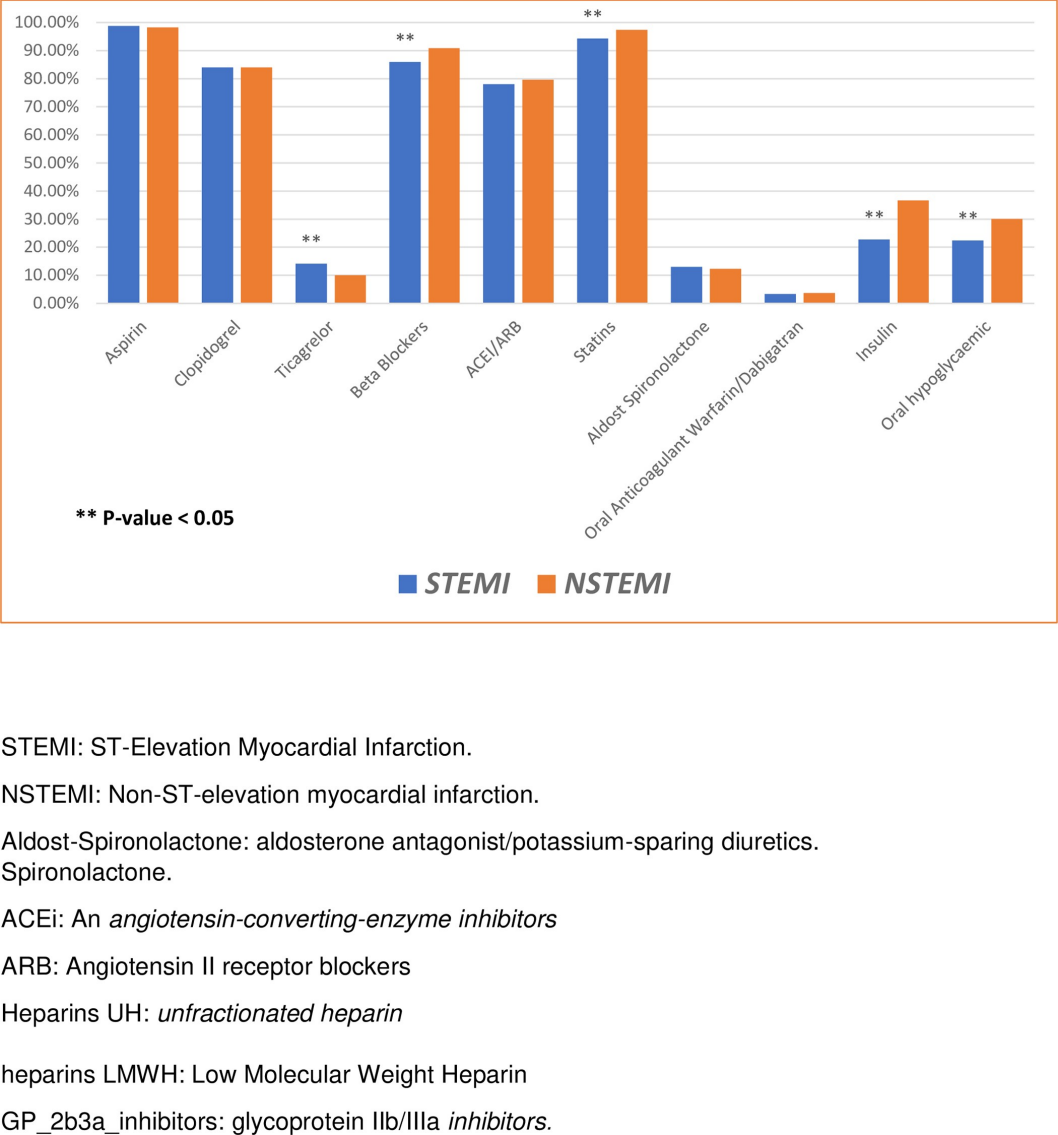
Medications use at hospital discharge for patients with acute myocardial infarctions. STEMI: ST-elevation myocardial infarction; NSTEMI: non-ST-elevation myocardial infarction.

These rates were also high at hospital discharge Fig 3: 96% of patients received aspirin, 92.4% received statins, 87% received beta-blockers, and 78.6% received angiotensin converting enzyme inhibitors or angiotensin receptor blockers (ACE-I/ARB).

Most patients received Clopidogrel (73.6%), compared to Ticagrelor (16.3%). Compared to patients with NSTEMI, patients with STEMI less frequently received beta-blockers (75% vs. 86%; p < 0.001) and ACE-I/ARBs (68% vs. 78%; p < 0.001).

### In-hospital procedures and outcomes

Evidence of left ventricular systolic dysfunction detected by echocardiography was found in 69.2% of all patients. Severe left ventricular systolic dysfunction (defined as < 30% ejection fraction) occurred in 10.5% of all patients. An elective coronary angiogram was performed in 44.2% of all patients, and only half the patients with NSTEMI received elective coronary angiography. Patients with NSTEMI were more likely to develop stroke, atrial fibrillation/flutter, recurrent myocardial infarction, or recurrent ischemia, compared to patients with STEMI (Table 2). In-hospital mortality occurred in 4.03% of patients, with no significant difference between STEMI and NSTEMI (4.1% and 3.9%, respectively; p = 0.87), and no significant difference between Saudis and non- Saudis (3.86% vs. 4.24%, respectively; p = 0.651). However, women had higher in-hospital mortality than men (7.2% vs. 3.5%, respectively; p = 0.001).

**Table 2:** In-hospital course and outcomes of patients with acute myocardial infarction.

Events	Total N = 2233	STEMI N = 1471 (65.88%)	NSTEMI N = 762 (34.12%)	P-value
Recurrent ischemia	196 (8.78%)	98 (6.66%)	98 (12.86%)	<0.001
Recurrent myocardial infarction	79 (3.54%)	31 (2.11%)	48 (6.30%)	<0.001
Atrial Fibrillation/Flutter	84 (3.76%)	40 (2.72%)	44 (5.77%)	<0.001
Heart Failure	255 (11.42%)	156 (10.61%)	99 (12.99%)	0.093
Cardiogenic Shock	150 (6.72%)	108 (7.34%)	42 (5.51%)	0.101
Ventricular tachycardia/ fibrillation	128 (5.73%)	84 (5.71%)	44 (5.77%)	0.951
Stroke	24 (1.07%)	10 (0.68%)	14 (1.84%)	0.012
Major bleeding	25 (1.12%)	19 (1.29%)	6 (0.79%)	0.283
Stent thrombosis	8 (0.50%)	5 (0.48%)	3 (0.54%)	0.877
Mortality	90 (4.03%)	60 (4.08%)	30 (3.94%)	0.872

Abbreviations: STEMI: ST-elevation myocardial infarction; NSTEMI: non-ST-elevation myocardial Infarction

Interestingly, the crude in hospital outcomes demonstrated that all outcomes were significantly higher in females but after adjudgment the only significant outcome was atrial fibrillation OR (95% CI) of 1.85(1.05–3.26) and P-value = 0.033 S5 Table. Similarly, when comparing in hospital outcomes for Saudis vs non Saudis there was no significant difference noted after adjustment. S7 Table.

### Follow-up

One month and 1-year follow-up data were collected for 1393 patients (65.0%). The rates of follow-up among Saudis were 88% and 80% at 1 month and 1year, respectively. Non-Saudis showed considerably lower follow-up rates of 34% at 1 month and 30% at 1 year. Re-admissions for the overall population occurred in 8.3% patients at 1 month and 19.2% at 1 year follow-ups, and they were mainly due to cardiac causes (84% at 1 month and 77% at 1 year). The cumulative all-cause mortality was 5.8% and 8.1% at 1 month and 1 year, respectively. Patients with NSTEMI and STEMI had similar mortality at 1 month, but those with NSTEMI had higher mortality at 1 year (7.0% vs. 3.8%, respectively; p = 0.014; S3 Table). Mortality was higher among women than men at 1 month (9.4% vs. 5.2%, p = 0.003) and at one year (12.2% vs. 7.4%; p = 0.004). The frequency of guideline-recommended oral treatments remained high at 1 year, with no significant difference between patients with STEMI and those with NSTEMI (S3 Fig and S4 Table). However, the use of Ticagrelor (16.3%) remained low compared to Clopidogrel (73.6%), with no significant difference between STEMI and NSTEMI groups.

At 1-year, 1180 patients (53%) were followed to assess optimal control of the CAD risk factors. Fasting blood glucose remained > 7 mmol/L in nearly 40% of patients with diabetes, and one quarter of all patients continued to smoke tobacco. The LDL-C level was greater than 2.5 mmol/L in 34% of patients, and systolic blood pressure was > 140 mmHg in 21% of patients.

## Discussion

The STARS-1 Program was the first survey of an AMI registry that was truly representative of the health care in Saudi Arabia. Unlike prior registry studies that we conducted in the past [2–4], the present study included almost all health care sectors from all geographic regions in the country. Furthermore, the snap-shot design enabled retrieval of high-quality data for consecutive patients with AMI from 50 hospitals; hence, we could reduce issues associated with missing cases and “registry-exhaustion”. Similar to results from prior ACS registry studies in Saudi Arabia, the present study demonstrated that patients were relatively young at presentation, had a high prevalence of CAD risk factors, and rarely utilized EMS. However, this STARS-1 Program study demonstrated several other unique findings. Nearly two thirds (66%) of patients with AMI had STEMI, compared to other registries in the developed countries for example, the Myocardial Ischemia National Audit Project UK MINAP data, of 155, 818 patients, 68,025 had STEMI (43.6%). [9]. The high prevalence of STEMI was probably due to the young average age of AMI presentation; our patients were nearly a decade younger than those studied in developed countries. Interestingly, the prevalence of STEMI was even higher in STARS than in previously studied local registries, most likely due to the higher proportion of non-Saudis (45% vs. 15%, respectively), which reflected the more representative sample derived from Ministry of Health hospitals. Non-Saudi patients with AMI were relatively younger, mostly males, and typically “blue-collar” workers. These individuals typically had relatively low access to advanced and life-saving tertiary-care services and had limited health care insurance policies [10]. Indeed, this study showed that non-Saudi patients with STEMI had significantly lower access to primary PCI compared to Saudis with STEMI (35.8% vs. 48.7%; respectively). Interestingly when comparing in- hospital outcomes among patients who underwent primary PCI for Saudi’s vs non-Saudi, there were no significant differences in either the crude or adjusted in-hospital outcomes. Nonetheless, the sample size is not large enough to have a meaningful comparison.

The rate of primary PCIs for patients with STEMI (42.5%) has increased significantly compared to rates reported in our previous registry studies (around 20%) [2–4]. This difference was most likely related to several factors, including the rise in the number of Cath Labs and interventional cardiologists in the country and the enhanced awareness of the benefits of primary PCI in the cardiac community. Nevertheless, the rate of primary PCIs in Saudi Arabia remained relatively low compared to western European countries. The Stent for Life initiative has demonstrated that the rate of primary PCIs was 23 per 1,000,000 inhabitants in Saudi Arabia, compared to 638 and 884 per 1,000,000 inhabitants in Germany and The Netherlands, respectively [11]. Our study also showed that there is a need for further improvements in STEMI care, because around 40% of patients with STEMI failed to achieve a door-to-balloon time of <90 min; failure to achieve this benchmark occurred significantly more frequently among women than men. Furthermore, women were less likely to receive thrombolytic therapy compared to men. Registry data in Switzerland and France also showed that primary PCI rates were lower for women than men (30.9% vs. 40.3%, and 47% vs. 55%, respectively) [12, 13]. These differences between the sexes were most likely related to multiple factors, including atypical symptoms, delay in seeking medical care, and under-referral of women to receive acute cardiac care and PCI procedures [14–17].

The decisions to provide a pharmaco-invasive approach (3%) and not to provide acute revascularization (30%) were mainly due to late presentations. These rates could be improved by enhancing the EMS infrastructure and EMS utilization, establishing organized STEMI programs and networks across the country, and enhancing the public awareness of AMI symptoms. The pharmaco-invasive approach is an effective alternative for patients that present with STEMI and are unlikely to receive timely access to a primary PCI. In the FAST-MI registry study in France, the time-to-reperfusion was most significantly shortened, when the patient was treated with fibrinolysis (median, 130 min), followed by rescue or elective PCI, compared to a primary PCI treatment (median, 300 min); but there was no significant difference in the long-term mortality between the two groups [18,19]. Furthermore, the Alberta Vital Heart Response STEMI Network in Canada implemented a pre-hospital triage system for determining whether a primary PCI or pharmaco-invasive treatment should be applied. In urban areas, approximately 57% of patients received primary PCIs, and 26% received pharmaco-invasive treatments. Conversely, in rural areas, approximately 23% of patients received primary PCIs, and 61% received pharmaco-invasive treatments. There was no difference in the composite outcome (death, reinfarction, congestive heart failure, and/or cardiogenic shock: 16.8% vs. 15.1%; p = 0.161) or major bleeding (7.9% vs. 8.0%; p = 0.951) between groups [20]. These results highlighted the practicality and the clinical benefits of establishing a well-organized STEMI network that enables the pharmaco-invasive approach, when it is not possible to perform a timely primary PCI. This network would be particularly beneficial in crowded major cities and in the under-served north and south regions of Saudi Arabia. (See Distribution of Cath Labs in Saudi Arabia, S4 Fig)

The rate of coronary revascularization also needs to be improved for patients with NSTEMI. In our group, about half of these patients did not receive a coronary angiogram. In addition, many hospitals were not enrolled into the registry, because the serum troponin test was not available. The implication of this latter finding could have a major impact on health care policy. It calls for action at the level of the Ministry of Health to implement the use of this essential diagnostic test in all hospitals that triage patients with suspected ACS [21].

This study was the first to investigate the rate of radial access in patients with AMI (51.6%) in a nation-wide registry in our region. We found that women were less likely than men to undergo this approach. Radial access was shown to be safe and effective for PCI in the study “Radial vs. Femoral Access for Coronary Angiography and Intervention in Patients with Acute Coronary Syndromes” (RIVAL study). They demonstrated that the rate of vascular complications was lower with radial access (1.4%) compared to femoral access (3.7%) [22]. Furthermore, these findings were recently substantiated in the Minimizing Adverse Hemorrhagic Events by Trans radial Access Site and Systemic Implementation of Angiox. (MATRIX) program [23]. That study demonstrated that the radial access group had less net adverse clinical events (15.2%) compared to the femoral access group (17·2%; RR = 0·87, CI: 0·78–0·97; p = 0·0128) [21].

The high frequency of guideline-recommended oral therapies that we observed was consistent with those reported in prior registry studies. However, in the STARS-1 Program, we demonstrated that Ticagrelor was used at low frequency in-hospital and after 1-year in patients with AMI. The use of Ticagrelor in AMI received a class-I recommendation from the American College of Cardiology and European Society of Cardiology [24, 25]. Potential causes for the low frequency of Ticagrelor use in our study included the higher cost, the need for twice daily dosing, and the side effects, primarily dyspnea. The Platelet inhibition and patient Outcomes (PLATO) trial, conducted in patients with ACS, reported that dyspnea occurred in 14.5% of the Ticagrelor group, compared to 8.7% of the Clopidogrel group; however, severe dyspnea occurred in only 0.4% and 0.3%, respectively [26]. Further studies are needed in our population to explore the reasons for such low use.

The overall in-hospital, 1-month, and 1-year mortality rates among our patients with AMI were 4%, 5.8%, and 8.1%; respectively. These rates were comparable to those reported for developed countries, but our patients presented at a relatively younger median age. Uncontrolled CAD risk factors were found in 20%-40% of our patients at the 1-year follow-up. This highlighted the need to develop optimal secondary prevention measures to be applied after hospital discharge.

Our study had some limitations. First, hospital enrollment was voluntary. Hence, there was a potential under-representation of patients in real-life clinical practice and patients in other non-enrolled hospitals, particularly hospitals with unavailable serum troponin testing. However, this limitation was mitigated by the enrollment of various health care sectors from all regions of the country. Second, a selection bias and the possibility of missing important unmeasured variables could have been introduced, due to the observational nature of registry studies. Third, only 14% of the 370 eligible hospitals were included in our study. This may indicate an overall small sample size. Nonetheless, all the Cath Lab hospitals enrolled in our registry were major tertiary health care centers that already have large “catchment area” and received referrals from many surrounding hospitals in their respective regions. Hence, we believe that the actual representation is much more than just 14%. Lastly, a large proportion of patients were missing at the 1 year follow-up, particularly among non-Saudis, mainly due to their poor access to health care [19]

In conclusion, the STARS-1 Program, with its unique study design, provided a representative snap-shot of the AMI care in Saudi Arabia. Our study design will be potentially helpful to other countries with large geographic areas. In Saudi Arabia, patients presented at relatively young age and had a high prevalence of CAD risk factors. Hence, there is a need for national primary prevention programs. In addition, we urgently need to improve the EMS infrastructure and utilization, establish an organized network of ACS programs, focus on timely acute revascularization, particularly for women, and improve access to health care for non-Saudis.

## Supporting information

S1 Fig Pie charts show (*left*) the numbers and types of health care sectors in Saudi Arabia, and (*right*) the proportions of Case Report Forms that were submitted from each sector. MOH: Ministry of Health hospitals, Private: Private Health Care hospitals, SFH: Security Forces Hospital, Military: Military hospitals, University: University hospital, National Guard: National Guard Hospital, CRF: Case Report Form.
(DOCX)

S2 Fig Pie charts show (*left*) the numbers and proportions of Cath Lab and non-Cath Lab hospitals enrolled in the registry, and (*right*) the proportions of Case Report Forms from each hospital type. Cath: heart catheterization equipment; CRF: case report form.
(DOCX)

S3 Fig Percentage of medication use at 1-year follow-up for patients with acute myocardial infarction. (DOCX)

S4 Fig Distribution of Catheterization Laboratories in Saudi Arabia. Paired numbers show the number of Catheterization Laboratories (first number) and the number of 24/7 Primary Percutaneous Coronary Intervention Catheterization Laboratories (second number) in Saudi Arabia in 2015–2016. (Source: Based on personal communications between the Principal Investigator (Prof. Khalid F AlHabib) and key-opinion leaders in Saudi Arabia in 2015/2016).
(DOCX)

S1 Table Reperfusion therapies in patients with STEMI: Comparison between men and women. (DOCX)

S2 Table Transport system and time lines for patients with acute STEMI that presented within 24 h of symptom onset; comparison between men and women. (DOCX)

S3 Table Readmission and mortality rates and their causes at 1-month and 1-year follow-ups for patients with acute ST-segment elevation and non-ST segment elevation myocardial infarctions (STEMI and NSTEMI, respectively). (DOCX)

S4 Table Medication use at 1-year follow-up for patients with acute ST-segment elevation and non-ST segment elevation myocardial infarction (STEMI and NSTEMI, respectively). (DOCX)

S5 Table Logistic regression, odds ratio adjusted gender. (DOCX)

S6 Table Logistic regression, odds ratio adjusted nationality. (DOCX)

S7 Table Table outcomes nationality (Saudi and Non-Saudi). (DOCX)

S1 File Co-investigators and data collectors. (DOCX)
