# Supplementary material for: The first survey of the Saudi Acute Myocardial Infarction Registry Program: Main results and long-term outcomes (STARS-1 Program)
Source: PLoS One. 2019 May 21;14(5):e0216551. doi: 10.1371/journal.pone.0216551 (PMC6528983; doi:10.1371/journal.pone.0216551)
Supplement: S1 Fig — Pie charts show (left) the numbers and types of health care sectors in Saudi Arabia, and (right) the proportions of Case Report Forms that were submitted from each sector. MOH: Ministry of Health hospitals, Private: Private Health Care hospitals, SFH: Security Forces Hospital, Military: Military hospitals, University: University hospital, National Guard: National Guard Hospital, CRF: Case Report Form. (DOCX) [file pone.0216551.s001.docx]

**S1 Fig.**Pie charts show (*left*) the numbers and types of health care sectors in Saudi Arabia, and (*right*) the proportions of Case Report Forms that were submitted from each sector. MOH: Ministry of Health hospitals, Private: Private Health Care hospitals, SFH: Security Forces Hospital, Military: Military hospitals, University: University hospital, National Guard: National Guard Hospital, CRF: Case Report Form

**
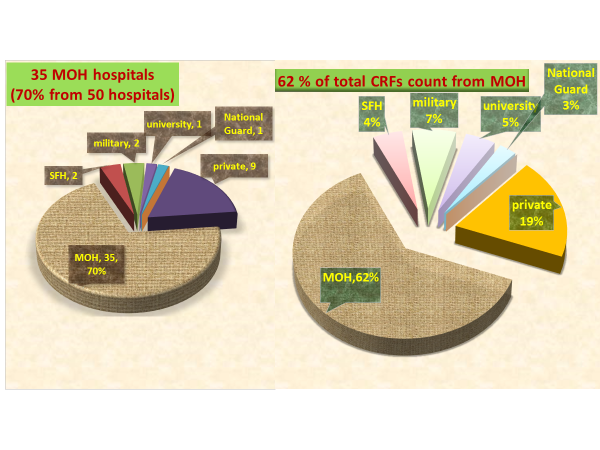
**
