# Supplementary material for: The first survey of the Saudi Acute Myocardial Infarction Registry Program: Main results and long-term outcomes (STARS-1 Program)
Source: PLoS One. 2019 May 21;14(5):e0216551. doi: 10.1371/journal.pone.0216551 (PMC6528983; doi:10.1371/journal.pone.0216551)
Supplement: S2 Fig — Pie charts show (left) the numbers and proportions of Cath Lab and non-Cath Lab hospitals enrolled in the registry, and (right) the proportions of Case Report Forms from each hospital type. Cath: heart catheterization equipment; CRF: case report form. (DOCX) [file pone.0216551.s002.docx]

**S2 Fig.** Pie charts show (*left*) the numbers and proportions of Cath Lab and non-Cath Lab hospitals enrolled in the registry, and (*right*) the proportions of Case Report Forms from each hospital type. Cath: heart catheterization equipment; CRF: case report form

**
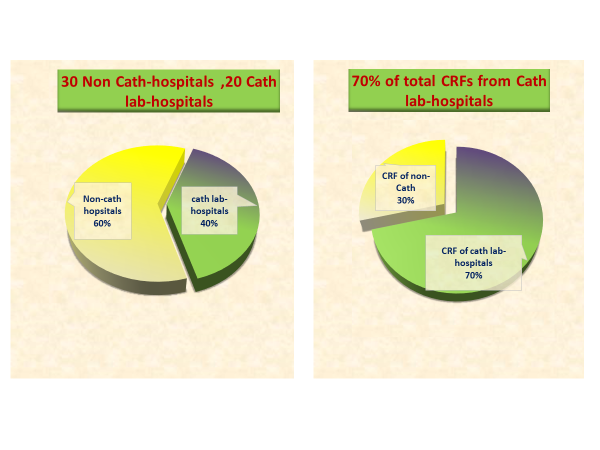
**
