# Supplementary material for: The first survey of the Saudi Acute Myocardial Infarction Registry Program: Main results and long-term outcomes (STARS-1 Program)
Source: PLoS One. 2019 May 21;14(5):e0216551. doi: 10.1371/journal.pone.0216551 (PMC6528983; doi:10.1371/journal.pone.0216551)

**S3 Fig.**Percentage of medication use at 1-year follow-up for patients with acute myocardial infarction


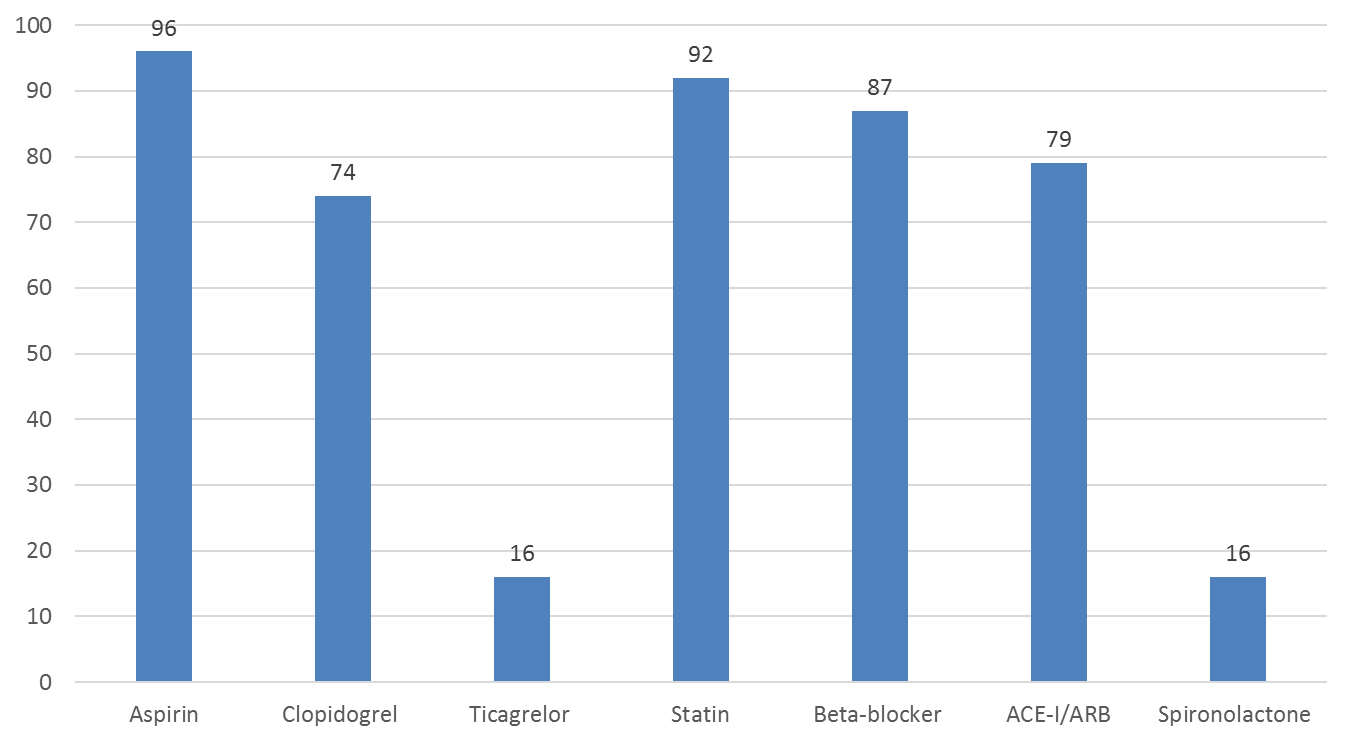

Supplement: S3 Fig — (DOCX) [file pone.0216551.s003.docx]
