# Supplementary material for: The first survey of the Saudi Acute Myocardial Infarction Registry Program: Main results and long-term outcomes (STARS-1 Program)
Source: PLoS One. 2019 May 21;14(5):e0216551. doi: 10.1371/journal.pone.0216551 (PMC6528983; doi:10.1371/journal.pone.0216551)

**S4 Fig.**  **Distribution of Catheterization Laboratories in Saudi Arabia.** Paired numbers show the number of Catheterization Laboratories (first number) and the number of 24/7 Primary Percutaneous Coronary Intervention Catheterization Laboratories (second number) in Saudi Arabia in 2015-2016. (Source: <http://viewer.nationalmap.gov/viewer/> .for illustrative purposes only.numbers Based on personal communications between the Principal Investigator (Prof. Khalid F AlHabib) and key-opinion leaders in Saudi Arabia in 2015/2016).


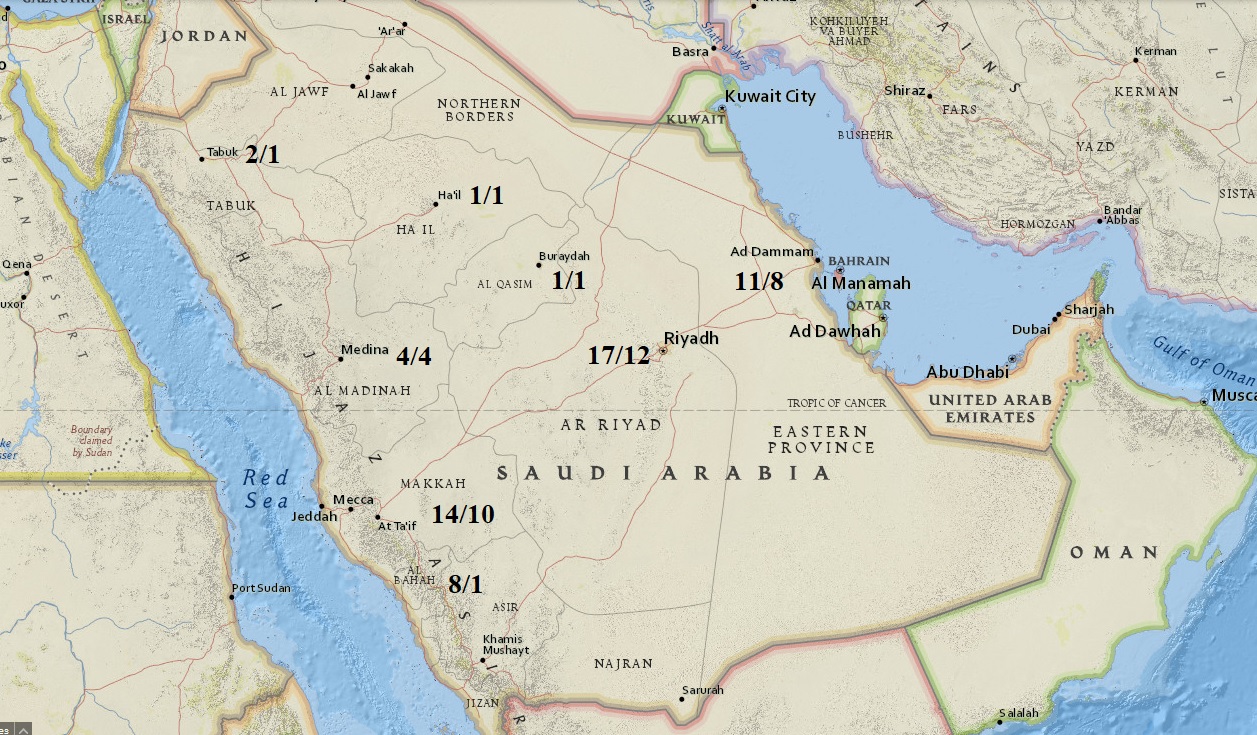

Supplement: S4 Fig — Paired numbers show the number of Catheterization Laboratories (first number) and the number of 24/7 Primary Percutaneous Coronary Intervention Catheterization Laboratories (second number) in Saudi Arabia in 2015–2016. (Source: Based on personal communications between the Principal Investigator (Prof. Khalid F AlHabib) and key-opinion leaders in Saudi Arabia in 2015/2016). (DOCX) [file pone.0216551.s004.docx]
