# Supplementary material for: The first survey of the Saudi Acute Myocardial Infarction Registry Program: Main results and long-term outcomes (STARS-1 Program)
Source: PLoS One. 2019 May 21;14(5):e0216551. doi: 10.1371/journal.pone.0216551 (PMC6528983; doi:10.1371/journal.pone.0216551)
Supplement: S1 Table — (DOCX) [file pone.0216551.s005.docx]

**S1 Table.
 Reperfusion therapies in patients with STEMI**: comparison between men and women

| **Therapy** | **Total**  **N=1471** | **Men**  **N=1315 (89.39%)** | **Women**  **N=156 (10.61%)** | P-value |
| --- | --- | --- | --- | --- |
| **Thrombolytic therapy** | **427 (29.03%)** | **398 (30.27%)** | **29 (18.59%)** | **0.002** |
| Thrombolytic therapy, but unsuccessful clinical reperfusion  Thrombolytic therapy, unsuccessful clinical reperfusion, and rescue PCI | 95 (22.3%)  36 (38%) | 89 (22.4%)  35 (39.3%) | 6 (20.7%)  1 (16.7%) | 0.834  0.123 |
| **Thrombolytic therapy and elective Cath/PCI or unsuccessful thrombolytic therapy and rescue PCI**  **(Pharmaco-invasive approach)** | **42 (2.86%)** | **41 (3.12%)** | **1 (0.64%)** | **0.079** |
| Primary PCI | 524 (35.62%) | 464 (35.29%) | 60 (38.46%) | 0.435 |
| **Treated with or transferred out for Primary PCI** | **625 (42.49%)** | **553 (42.05%)** | **72 (46.15%)** | **0.327** |
| Vascular access in patients treated with Primary PCI | | | | |
| Femoral | 267 (51.64%) | 227 (49.35%) | 40 (70.18%) | 0.012 |
| Radial | 249 (48.16%) | 232 (50.43%) | 17 (29.82%) |  |
| Brachial | 1 (0.19%) | 1 (0.22%) | 0 (0.00%) |  |
| Thrombectomy device in Primary PCI | 94 (18.65%) | 88 (19.56%) | 6 (11.11%) | 0.132 |
| **NOT treated with thrombolytic therapy or Primary PCI or transferred out for primary PCI (no reperfusion)** | **419 (28.48%)** | **366 (27.83%)** | **55 (33.97%)** | **0.051** |
| Reasons for no thrombolytic therapy or primary PCI | | | | |
| Late presentation | 184 (52.57%) | 160 (52.46%) | 24 (53.33%) | 0.187 |
| Missed | 3 (0.86%) | 2 (0.66%) | 1 (2.22%) |  |
| Contraindication | 25 (7.14%) | 19 (6.23%) | 6 (13.33%) |  |
| Other | 138 (39.43%) | 124 (40.66%) | 14 (31.11%) |  |
