# Supplementary material for: The first survey of the Saudi Acute Myocardial Infarction Registry Program: Main results and long-term outcomes (STARS-1 Program)
Source: PLoS One. 2019 May 21;14(5):e0216551. doi: 10.1371/journal.pone.0216551 (PMC6528983; doi:10.1371/journal.pone.0216551)
Supplement: S2 Table — (DOCX) [file pone.0216551.s006.docx]

**S2 Table.
 Transport system and time lines for patients with acute STEMI that presented within 24 h of symptom onset**; comparison between men and women

| **Treatment factors** | **Total**  **N=1178** | **Men**  **N=1066 (90.49%)** | **Women**  **N=112 (9.51%)** | **P-value** |
| --- | --- | --- | --- | --- |
| Transferred by Saudi Red Crescent | 61 (5.18%) | 53 (4.97%) | 8 (7.14%) | 0.324 |
| Evaluated by FMC before arriving at hospital | 399 (33.87%) | 363 (34.05%) | 36 (32.14%) | 0.685 |
| Visited an ED | 332 (28.18%) | 300 (28.14%) | 32 (28.57%) | 0.923 |
| Visited a Clinic/Doctor | 84 (7.13%) | 80 (7.51%) | 4 (3.57%) | 0.173 |
| Visited a Pharmacy | 3 (0.25%) | 3 (0.28%) | 0 (0.00%) | 0.595 |
| Visited an ED after a FMC | 332 (83.21%) | 300 (82.64%) | 32 (88.89%) | 0.339 |
| Visited a Clinic/Doctor after a FMC | 84 (21.05%) | 80 (22.04%) | 4 (11.11%) | 0.125 |
| Visited a Pharmacy after a FMC | 3 (0.75%) | 3 (0.83%) | 0 (0.00%) | 0.584 |
| Symptoms to FMC, min; Median (IQR) | 110.0 (199.5) | 105.0 (200.0) | 125.0 (180.0) | 0.219 |
| Symptom to FMC in patients treated with thrombolysis, min; Median (IQR) | 60.00 (222.0) | 60.00 (192.0) | 590.0 (0.00) “only 1 patient!” | 0.140 |
| Symptom to FMC in patients treated with Primary PCI, min; Median (IQR) | 106.0 (168.0) | 102.5 (168.0) | 125.0 (150.0) | 0.630 |
| FMC to ED: All Hospitals, min; Median (IQR) | 155 (280.0) | 151 (293.0) | 204 (199.0) | 0.981 |
| FMC to ED: Non-Cath Lab Hospitals, min;, Median (IQR) | 152 (21.0) | 137 (219.0) | 250 (677.0) | 0.011 |
| FMC to ED: Cath Lab Hospitals, min; Median (IQR) | 164.0 (295.0) | 164.0 (315.0) | 147.5 (198.0) | 0.441 |
| Symptom to ED arrival |  |  |  |  |
| < 3 h | 580 (49.24%) | 521 (48.87%) | 59 (52.68%) | 0.742 |
| 3-12 h | 459 (38.96%) | 418 (39.21%) | 41 (36.61%) |  |
| 12-24 h | 139 (11.80%) | 127 (11.91%) | 12 (10.71%) |  |
| Symptom to ED arrival: All Hospitals, min; Median (IQR) | 180.0 (310.0) | 180.0 (314.0) | 161.0 (288.0) | 0.409 |
| Symptom to ED arrival: Cath Lab Hospitals, min; Median (IQR) | 217.0 (330.0) | 215.0 (335.0) | 235.0 (272.0) | 0.888 |
| Symptom to ED arrival: Non-Cath Lab Hospitals, min; Median (IQR) | 120.0 (251.0) | 120.0 (253.0) | 100.0 (191.0) | 0.557 |
| Door to ECG, min; Median (IQR) | 9.00 (8.00) | 9.00 (8.00) | 10.00 (8.50) | 0.222 |
| Door to ECG < 10 min; n (%) | 599 (50.85%) | 546 (51.22%) | 53 (47.32%) | 0.432 |
| Door to Needle, min; Median (IQR) | 30.00 (35.00) | 30.00 (35.00) | 28.00 (31.00) | 0.224 |
| Door to Needle < 30 min; n (%) | 188 (44.87%) | 174 (44.39%) | 14 (51.85%) | 0.451 |
| Door to Needle < 30 min: Cath Lab hospitals; n (%) | 75 (42.86%) | 73 (42.94%) | 2 (40.00%) | 0.895 |
| Door to Needle < 30 min: Non-Cath Lab hospitals; n (%) | 113 (46.31%) | 101 (45.50%) | 12 (54.55%) | 0.416 |
| FMC to needle, min; Median (IQR) | 140.0 (227.0) | 135.0 (227.0) | 857.5 (1245) | 0.121 |
| Door to Balloon, min; Median (IQR) | 75.00 (84.00) | 73.00 (74.50) | 103.0 (196.0) | 0.033 |
| Door to Balloon < 90 min; n (%) | 290 (62.63%) | 270 (64.90%) | 20 (42.55%) | 0.003 |
| FMC to Balloon: Cath Lab hospitals, min; Median (IQR) | 210.0 (347.0) | 206.0 (340.0) | 307.0 (419.0) | 0.316 |

FMC: first medical contact; ED: Emergency department; IQR: interquartile range; PCI: percutaneous cardiac intervention; Cath: cardiac catheterization equipment; ECG: electrocardiogram;
