# Supplementary material for: The first survey of the Saudi Acute Myocardial Infarction Registry Program: Main results and long-term outcomes (STARS-1 Program)
Source: PLoS One. 2019 May 21;14(5):e0216551. doi: 10.1371/journal.pone.0216551 (PMC6528983; doi:10.1371/journal.pone.0216551)
Supplement: S3 Table — (DOCX) [file pone.0216551.s007.docx]

**S3 Table.
Readmission and mortality rates** and their causes at 1-month and 1-year follow-ups for patients with acute ST-segment elevation and non-ST segment elevation myocardial infarctions (STEMI and NSTEMI, respectively).

| **Outcome** | **Total**  **N=1393** | **STEMI**  **N=885 (63.53%)** | **NSTEMI**  **N=508 (36.47%)** | **P- value** |
| --- | --- | --- | --- | --- |
| One-month readmission | 115 (8.26%) | 68 (7.68%) | 47 (9.25%) | 0.306 |
| One-month readmission cause | | | | |
| Cardiac Cause | 96 (84.96%) | 56 (83.58%) | 40 (86.96%) | 0.622 |
| Non-cardiac Cause | 17 (15.04%) | 11 (16.42%) | 6 (13.04%) |  |
| One-month mortality | 39 (2.78%) | 22 (2.47%) | 17 (3.30%) | 0.362 |
| One-month mortality cause | | | | |
| Cardiac Cause | 26 (70.27%) | 15 (71.43%) | 11 (68.75%) | 0.860 |
| Non-cardiac Cause | 11 (29.73%) | 6 (28.57%) | 5 (31.25%) |  |
| One-year readmission | 232 (19.24%) | 135 (17.88%) | 97 (21.51%) | 0.122 |
| One-year readmission cause | | | | |
| Cardiac Cause | 168 (77.78%) | 99 (77.34%) | 69 (78.41%) | 0.853 |
| Non-cardiac Cause | 48 (22.22%) | 29 (22.66%) | 19 (21.59%) |  |
| One-year mortality | 56 (4.96%) | 27 (3.76%) | 29 (7.06%) | 0.014 |
| One-year mortality cause | | | | |
| Cardiac Cause | 48 (85.71%) | 23 (85.19%) | 25 (86.21%) | 0.913 |
| Non-cardiac Cause | 8 (14.29%) | 4 (14.81%) | 4 (13.79%) |  |
