# Supplementary material for: The first survey of the Saudi Acute Myocardial Infarction Registry Program: Main results and long-term outcomes (STARS-1 Program)
Source: PLoS One. 2019 May 21;14(5):e0216551. doi: 10.1371/journal.pone.0216551 (PMC6528983; doi:10.1371/journal.pone.0216551)
Supplement: S4 Table — (DOCX) [file pone.0216551.s008.docx]

**S4 Table**. **Medication use at 1-year follow-up for patients with** **acute ST-segment elevation and non-ST segment elevation myocardial infarction (STEMI and NSTEMI, respectively).**

| **Medication** | **STEMI**  **N=769 (64.68%)** | **NSTEMI**  **N=420 (35.32%)** | **P-value** |
| --- | --- | --- | --- |
| Aspirin | 714 (95.58%) | 394 (96.57%) | 0.417 |
| Clopidogrel | 528 (71.16%) | 315 (78.16%) | 0.010 |
| Ticagrelor | 117 (15.79%) | 70 (17.33%) | 0.501 |
| Statin | 686 (92.33%) | 378 (92.65%) | 0.845 |
| Beta blocker | 636 (85.83%) | 361 (89.14%) | 0.112 |
| ACEI/ARB | 586 (78.98%) | 315 (77.78%) | 0.637 |
| Spironolactone | 113 (15.25%) | 65 (16.05%) | 0.721 |
| Oral anticoagulant | 15 (2.02%) | 8 (1.98%) | 0.955 |

ACEI/ARB: angiotensin converter enzyme inhibitor/angiotensin receptor blocker
