# Supplementary material for: The first survey of the Saudi Acute Myocardial Infarction Registry Program: Main results and long-term outcomes (STARS-1 Program)
Source: PLoS One. 2019 May 21;14(5):e0216551. doi: 10.1371/journal.pone.0216551 (PMC6528983; doi:10.1371/journal.pone.0216551)
Supplement: S5 Table — (DOCX) [file pone.0216551.s009.docx]

**S5 Table.**
 **Logistic Regression, Odds Ratio adjusted Gender.**

| **Covariate** | **Level** | **Crude OR(95% CI)** | **P-value** | **Adjusted OR (95% CI)** | **P-value** |
| --- | --- | --- | --- | --- | --- |
| Mortality | Female | 2.13(1.308,3.48) | 0.002 | 1.42(0.733,2.74) | 0.300 |
| Recurrent Ischemia | Female | 1.61(1.112,2.33) | 0.012 | 1.20(0.766,1.89) | 0.422 |
| Heart Failure | Female | 2.14(1.564,2.93) | < 0.001 | 1.51(0.982,2.32) | 0.061 |
| Cardiogenic Shock | Female | 1.62(1.069,2.45) | 0.023 | 1.17(0.633,2.16) | 0.617 |
| Stroke | Female | 2.49(1.026,6.06) | 0.044 | 1.59(0.546,4.61) | 0.396 |
| Major bleeding | Female | 2.35(0.975,5.68) | 0.057 | 1.25(0.401,3.90) | 0.700 |
| Recurrent MI | Female | 2.25(1.344,3.76) | 0.002 | 1.38(0.718,2.64) | 0.336 |
| Atrial Fibrillation/Flutter | Female | 3.00(1.868,4.82) | < 0.001 | 1.85(1.053,3.26) | 0.033 |
| VT/VF arrest | Female | 1.65(1.062,2.57) | 0.026 | 1.34(0.768,2.32) | 0.305 |
