# Supplementary material for: The first survey of the Saudi Acute Myocardial Infarction Registry Program: Main results and long-term outcomes (STARS-1 Program)
Source: PLoS One. 2019 May 21;14(5):e0216551. doi: 10.1371/journal.pone.0216551 (PMC6528983; doi:10.1371/journal.pone.0216551)
Supplement: S6 Table — (DOCX) [file pone.0216551.s010.docx]

**S6 Table.**
 **Logistic Regression, Odds Ratio adjusted Nationality**.

| **Covariate** | **Level** | **Crude OR(95% CI)** | **P-value** | **Adjusted OR (95% CI)** | **P-value** |
| --- | --- | --- | --- | --- | --- |
| Mortality | Saudi | 0.91(0.595,1.38) | 0.652 | 0.84(0.359,1.95) | 0.678 |
| Recurrent Ischemia | Saudi | 1.35(1.00,1.82) | 0.050 | 1.50(0.839,2.69) | 0.171 |
| Heart Failure | Saudi | 1.33(1.022,1.74) | 0.034 | 1.39(0.809,2.39) | 0.233 |
| Cardiogenic Shock | Saudi | 1.13(0.808,1.58) | 0.478 | 1.33(0.616,2.86) | 0.470 |
| Stroke | Saudi | 3.20(1.191,8.60) | 0.021 | 3.75(0.475,29.55) | 0.210 |
| Major bleeding | Saudi | 1.25(0.561,2.80) | 0.583 | 3.15(0.379,26.16) | 0.288 |
| Recurrent MI | Saudi | 2.22(1.345,3.65) | 0.002 | 1.17(0.492,2.77) | 0.726 |
| Atrial Fibrillation/Flutter | Saudi | 3.17(1.871,5.38) | < 0.001 | 2.42(0.921,6.34) | 0.073 |
| VT/VF arrest | Saudi | 1.19(0.829,1.71) | 0.344 | 1.82(0.861,3.85) | 0.117 |
