# Supplementary material for: The first survey of the Saudi Acute Myocardial Infarction Registry Program: Main results and long-term outcomes (STARS-1 Program)
Source: PLoS One. 2019 May 21;14(5):e0216551. doi: 10.1371/journal.pone.0216551 (PMC6528983; doi:10.1371/journal.pone.0216551)
Supplement: S7 Table — (DOCX) [file pone.0216551.s011.docx]

**S7 Table.**
 **Table Outcomes Nationality (Saudi and Non-Saudi).**

| **Outcomes** | **Total**  **N=524** | **Saudi Arabia**  **N=303** | **Non-Saudi**  **N=221** | **P-value** |
| --- | --- | --- | --- | --- |
| Recurrent ischemia | 8 (1.53%) | 4 (1.32%) | 4 (1.81%) | 0.652 |
| Recurrent MI | 4 (0.76%) | 3 (0.99%) | 1 (0.45%) | 0.485 |
| Atrial Fibrillation/Flutter | 12 (2.29%) | 10 (3.30%) | 2 (0.90%) | 0.070 |
| Heart Failure | 51 (9.73%) | 29 (9.57%) | 22 (9.95%) | 0.884 |
| Cardiogenic Shock | 51 (9.73%) | 24 (7.92%) | 27 (12.22%) | 0.101 |
| VT/VF arrest | 34 (6.49%) | 20 (6.60%) | 14 (6.33%) | 0.903 |
| Stroke | 4 (0.76%) | 4 (1.32%) | 0 (0.00%) | 0.086 |
| Major bleeding | 10 (1.91%) | 7 (2.31%) | 3 (1.36%) | 0.431 |
| Stent thrombosis | 3 (0.57%) | 1 (0.33%) | 2 (0.90%) | 0.391 |
| Mortality | 20 (3.82%) | 9 (2.97%) | 11 (4.98%) | 0.236 |
