# Supplementary material for: The first survey of the Saudi Acute Myocardial Infarction Registry Program: Main results and long-term outcomes (STARS-1 Program)
Source: PLoS One. 2019 May 21;14(5):e0216551. doi: 10.1371/journal.pone.0216551 (PMC6528983; doi:10.1371/journal.pone.0216551)
Supplement: S1 File — (DOCX) [file pone.0216551.s012.docx]

**S1 File.**

**Co-investigators and data collectors**

**Riyadh** Khalid F. AlHabib, Hussam Alfaleh, Ahmad Hersi, Tarek Kashour, Mohammad Ali, Hani B Altaradi, Hassan Mhish MD, Abdulrahman Nouri Abdo, Fawaz Almutairi, Mohammed R. Arafah, Raed AlKutshan, Mubarak Aldosari, Basel Y. AlSabatien, Mohammad Alrazzaz, Adel M. Maria, Aziza H. Aref, Amr D.Sharafi, Nour Alkamel, Bob Cardinal, Samih R. Lawand, Mohamed zeyad Mohamed, Hoyam A.Abdoun, Omar Alnobani, ElMunzer Mahgoub, Asia, Abdulrahman Alqahtani, **Makkah**  Abdulhalim J.kinsara, Muhammed M.Selim,Ayman M. Morsy , Fathi A. AlTohar,Ammar A. Alrifai, Awatif A. Awaad, Hassan El-Sayed, Yasir Ismail, Nader AlMasry, Zainah Ashlie Y.Chung, Owais Ahmad Halabi, suryakant Pankaj, **Madinah** Saleh Alghamdi, Sherief Mansour, Ashraf A. Atwa,Salah Abdelkader, Naif Altamimi, Elnatheer Saleh, Wael Alhaidari, Saleh Mayyas, **Eastern Province** Shukri AlSaif, El Husseini A. ElShihawy, Ali H. Busaleh, Mohammed Abdalmoutaleb, Essam M. Fawzy, Hind Abdulrahman Alkammar, Attiea M. Saleh, Aamir Shahzad, Ahmad Soliman, Ms. Robelyn C. Corbita, Amro Elgamri, Musaab Zaroug., **Qassim** Hassan Khalaf MD, Zaki Mokhtar, Adil M. Saleh, Mohammed A. Ahmad, Mohammad I. khok, Perna Ditte Laboriante, Abdalrahman Albadrany , **Asir** Mushabab Al-Murayeh, Adel Almasswary, Mohammed Alshehri, Khalid M. Abohatab, Turki AlGarni, Zia-Ul-Sabah, Anwar Jafri, Juli N.Kunjumon., **Tabuk** Gamal Abdin Hussein, Modaser Butt, Hassan Darwish., **Northern Borders** Ibrahim Altaj, Farhan Abdullah, Helmi Abdulaziz, Mohammed Abdulaziz, farhan Hassan Abdullah , **Najran** Yahya Alhosni, Ibrahim Hassan. **Jawf** Hadia B.Osman, Najeebullah Bugti, Atif A. Aziz, Manzoor Memon, Shahima**Al-Bahah** Abdulrahman Alarabi.**Jizan** Ibrahim A. AlHarbi.**Ha'il** Hassan Khalaf MD, Moataz A. ElSanan.
